# Perceived compassionate care and associated factors among patients with mental illness at Tibebe Ghion specialized and Felege Hiwot comprehensive specialized hospital, Northwest Ethiopia

**DOI:** 10.1186/s12913-023-09665-4

**Published:** 2023-06-17

**Authors:** Agmas Wassie Abate, Melak Menberu, Habte Belete, Temesgen Ergetie, Assefa Agegnehu Teshome, Aklile Tsega Chekol, Mastewal Aschale, Wondimnew Desalegn

**Affiliations:** 1 Department of Psychiatry, Dr. Ambachew Memorial Hospital, Amhara Regional Health Bureau, Tach Gaynt, Ethiopia; 2 Department of Psychiatry, College of Medicine and Health Sciences, Bahir Dar University, Bahir Dar, Ethiopia; 3 Department of Biomedical Science, College of Health Science, Debre Tabor University, Debre Tabor, Ethiopia; 4 Department of Psychiatry, College of Health Science, Hawassa University, Hawassa, Ethiopia; 5 Department of Public Health, College of Health Science, Debre Tabor University, Debre Tabor, Ethiopia

**Keywords:** Compassionate care, Mental health service, Associated factors, Patients, Ethiopia

## Abstract

**Background:**

Compassion is the first ethical principle of health care to provide high- quality care that influences patient satisfaction and treatment outcome. However, there is limited data on the level of compassionate mental health care practice in low-resource countries like Ethiopia.

**Objectives:**

This study aimed to assess the level of perceived compassionate care and associated factors among patients with mental illness at Tibebe Ghion specialized and Felege Hiwot comprehensive specialized hospital, North West, Ethiopia, 2022.

**Methods:**

An institutional-based cross-sectional study design was conducted from June 18 to July 16, 2022, at Tibebe Ghion Specialized and Felege Hiwot Comprehensive Specialized Hospital. A systematic random sampling technique was used. The level of perceived compassionate care was assessed by the validated 12-item Schwartz Center Compassionate Care Scale among 423 patients with mental illness. Epicollect-5 was used to collect data, which was then exported to the Statistical Product and Service solution version 25 for analysis. Variables with a *P*-value < 0.05, and 95% confidence interval (CI) were used to declare significant variables at the multivariate logistic regression analysis.

**Result:**

The level of perceived good compassionate care was 47.5% (95% CI 42.6%-52.4%). Factors including urban residence (AOR = 1.90; 95%CI 1.08–3.36), duration of illness < 24 months (AOR = 2.68; 95% CI 1.27–5.65), strong social support (AOR = 4.43; 95%CI 2.16–9.10), shared decision making (AOR = 3.93; 95% CI 2.27–6.81), low perceived stigma(AOR = 2.97; 95% CI 1.54–5.72) and low patient anticipated stigma (AOR = 2.92; 95% CI 1.56–5.48) were positively associated with good compassionate care.

**Conclusion and recommendation:**

Less than half of the patients had received good compassionate care. Compassionate mental health care needs public health attention. Policymakers should emphasize on compassionate care continuity by including it in the health care curriculum and design appropriate policies to strengthen compassionate care.

**Supplementary Information:**

The online version contains supplementary material available at 10.1186/s12913-023-09665-4.

## Introduction

According to international research, compassion has been considered as an important human value for millennia. However, only in the last few decades, compassion has developed a focus of interest in scientific research. More recently, the field of medicine has seen an advance in interest in compassion as a key aspect of person-centered healthcare [1].

Perceived compassionate care is a state of apprehension for the suffering or unmet needs of another, coupled with a desire to improve that suffering [2]. A growing number of programs aim to improve self-care and compassion for others [3]. Over the past 10 years, there has been an improved focus in research, policy, and practice on the public health importance of compassion in health care, but lack of evidence on compassion and compassionate mental health care in patients with mental illness [4]. According to WHO report, compassion is essential for quality health care whereas lack of quality health care is a leading cause of death of approximately 8.6 million deaths per year [5]. Of these, 5 million occur in patients who are able to access the health system but receive poor quality care [5]. The level of compassionate care in health services ranges from 38.8% to 72.8% including 38.8% in North Showa Ethiopia [6], 51.6% in North East Ethiopia [7], 53% in USA [8], 57% in Addis Ababa [9], 58 in USA [8] and 72.8% in North East Ethiopia [10]. Compassionate care (CC) is a mainstay for cultivating health-seeking behavior: It has been given much consideration globally; following the alarms that healthcare often falls significantly. However, less research weight has been paid, and no sufficient data in low-income countries including Ethiopia [10]. Compassion mental health care is the center of focus for patients, families, and healthcare providers as a key element of high-quality mental health care. Good mental health care is associated with high performance, high achievement, and the ability to cope with stress [11]. Compassion is a central pillar in Ethiopia’s new Health Sector Transformation Plan (HSTP), which lays out a strategy to improve the quality and access to mental health services [5]. However, no evidence on the level of compassionate care and its determinant factors on mental health care. It is essential to provide data on level of compassionate care as the quality health services and the drive towards universal health coverage is recognized by the World Health Organization (WHO), notably in Ethiopia [5, 12]. From previous studies, factors that were affecting level of patient compassionate care in mental health services were socio-demographic, clinical, psychosocial, and service-related factors [13, 14]. Staff working in acute inpatient mental health wards face high levels of stress and fatigue, which can impact level of compassionate health care [15].

Bulletin of the WHO demonstrated that benefits of CC, including improved patient satisfaction, increased treatment adherence and improved health outcomes. The modern style to mental health care seeks to engage attention of both patients and public in developing quality of mental health care services by so long as compassionate care for patients’ with mental illness.

Even though, patients who had got good compassionated care are more likely to seek treatment and ought to have a better outcome as compared to psychiatric treatment without compassionate care, assessing compassionate mental health care was not understood. Assessment of CC at outpatient psychiatry department in specialized hospitals has great importance to improve and change service delivery focusing on identifying factors that affects CC with mental health services. It will help to increase treatment-seeking behavior of people.

In addition, there was no similar research completed in Ethiopia. It helps as a source of data/information for stakeholders and researchers who work in the area of mental health service and those who will conduct further studies on the issue under inquiry. Therefore, the aim of this study is to see the level of CC at the outpatient department of psychiatric clinic and identifying factors that affect CC with mental health services.

## Methods and material

### Study area and period

The study was conducted at specialized hospitals in Bahir Dar city, Ethiopia. Tibebe Ghion Specialized hospital (TGSH) gives mental health services with four outpatient departments, one emergency room, and two inpatients with a total bed of 13. It also gives psychotherapy services. The community is served by two psychiatrists, seven mental health professional specialists (MSc), one MSc in clinical counseling, and six psychiatric professionals. The total number of annual outpatient clients was four thousand eight hundred sixty four (four hundred five per month based on the monthly report of the psychiatry unit). Felege Hiwot Comprehensive specialized (FHCSH) also provides mental health services with inpatients (Seventeen beds) and four outpatient departments. It serves a total of nineteen thousands two hundred (19,200) clients annually (One thousands six hundred per month). The patient was served by four mental health professional specialists (MSc) and six psychiatry professionals. The monthly patient flow for both hospitals in psychiatry outpatient was Two thousand five patients. The study was conducted from June 18/2022 to July 18, 2022.

### Study design and population

An institutional-based cross-sectional study design was used. All patients who visited the outpatient department (OPD) for mental health services at TGSH and FHCSH and who were available at the time of data collection and whose age was 18 years or older were included. Those patients who were seriously ill and had no insight were excluded. Patient with major neurocognitive disorder (dementia) and with neurological disorder were not included in the study.

### Sample size determination

Sample size was calculated by using the previous report on compassionate and respectful care, 51.55% in Ethiopia [7]. By using a single population proportion formula with a 95% confidence interval and a 5% margin of error, the sample size was calculated by.

Adding a 10% non-response rate, the final sample size was 423.

**Fig. 1:**
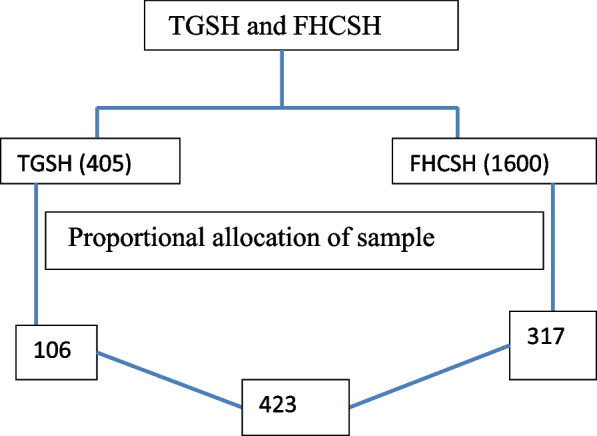
Schematic presentation of proportional allocation of sample among patients with mental illness TGSH and FHCSH, North West, Ethiopia, 2022

To get representative data in both hospitals, proportional allocation of the sample was calculated based on patient flow per month, and then the proportion would be 1:4. The final sample size distribution was 106 for TGSH and the rest 317 for FHCSH (Fig. 1).

### Sampling technique and procedure

A systematic random sampling was applied to recruit the study samples. As the systemic random sampling method needs to determine the sampling interval (k) value, and then calculate K as K = N/n = 2005/423 = 4. Then, the first participant was selected by the lottery method and then the remaining samples were selected every 4 patients (Fig. 1).

### Data collection methods and tools

The questionnaire consists of structured interviewer-administered questions including 1) socio-demographic, 2) clinical factors, 3) level of perceived compassionate care 4) social support, 5) shared decision making, 6) perceived stigma, and 7) patient anticipated stigma towards health workers. Four BSc psychiatric nurses collected the data by using Epicollect-5 software, and supervised by two psychiatry unit coordinators, one from each of hospitals.

The level of perceived compassionate care was measured by a validated 12-item Schwartz Center Compassionate Care Scale® (SCCCS) [16]. Each item is scored on a 10-point scale from 1–10 with response options that range from 1 (not at all successful) to 10 (very successful). A total perceived compassionate care score was calculated, and a total mean was computed (76.74) and it was considered as cutoff point. A score greater than 76.74 were considered as good perceived compassionate care, and less than 76.74 was considered as a poor compassionate care. Permission to use the scale was granted by Beth A. Lown, the copyright holder of the instrument, and the English and Amharic versions were obtained from Merkeb Zeray, the validator of the tool in Ethiopia [17]. The tool was validated in Ethiopia and showed internal consistency (a = 0.95) with item-to-total correlations that were excellent, ranging from 0.83 to 0.93, and a test–retest reliability of 0.90 [6, 16–19].

Social support was assessed by using the Oslo Social Support Scale (OSSS) — 3-item and has Cronbach’s alpha of 0.75 and a range value of 3–14. The level of score was further categorized as "Poor support," 3–8; "moderate support," 9–11; and "strong support," 12–14, with which internal consistency could be regarded as acceptable at α = 0.640 [20].

The 9-item Shared Decision Making Questionnaire (SDM-Q-9) was used to assess the decision-making style of a physician on patient treatment with Cronbach’s value of = 0.938 [21]. Summing up all items leads to a raw total score between 0 and 45, this was then transformed to 0 to 100 by multiplying 20/9. The mean was computed and used as a cut point: agree > 54.48 and disagree < 54.48 [21].

The level of perceived stigma was measured by the Stigma Scale for receiving psychological help, which has a five-item scale with Cronbach’s alpha of 0.74 and score was ranging from 0, lowest perceived stigma, to 15, highest perceived stigma [22]. A score above 6.86 was considered as having high perceived stigma and a score below 6.68 was considered as having a low perceived stigma [22].

Patient anticipated stigma from health workers: -measured by the patient anticipated stigma to health care workers scale, a four-item Likert scale ranging from 1 (very unlikely) to 5 (very likely) (Cronbach’s = 0:83) [23]. The mean score above 10.89 was regarded as a high patient anticipated stigma to health workers, while a score below 10.89 was regarded as a low patient anticipated stigma to health workers [23].

### Operational definitions

#### Perceived compassionate care

It is critical to high-quality health care and was measured by the Schwartz Center Compassionate Care Scale® (SCCCS), which assesses the level of perceived compassionate health care and categorizes it as follows:

- Good:—A score greater than the mean (76.74) considered good compassionate care
- Poor:—Score less than the mean (76.74) considered poor compassionate care [5, 19, 24].

#### Social support

Measured by OSLO 3 social support which is categorized into:

- Poor social support—3–8
- Moderate social support—9–11
- Strong social support- 12–14 [25].

#### Shared decision-making

This is measured by the core instrument, which consists of nine items, which can be rated on a six-point scale from ‘‘completely disagree’’ (0) to ‘‘completely agree’’ [5]. Summing up all items leads to a raw total score between 0 and 45, this was then transformed to 0 to 100 by multiplying 20/9. The mean was computed and used as a cut point: agree > 54.48 and disagree < 54.48 [26].

#### Perceived stigma

The Stigma Scale for Receiving Psychological Help, a five-item scale with an internal Cronbach’s alpha of 0.74, was created by summing the scores (0–3) for each item, ranging from 0, the lowest perceived stigma, to 15, the highest perceived stigma; then the cut point is 6.86. The score above 6.86 was considered as having high perceived stigma and the score below 6.68 considered as having a low perceived stigma [20].

#### Patient anticipated stigma from health workers

A four-item survey with a Likert scale ranging from 1 (very unlikely) to 5 (very likely) is used to assess patient anticipated stigma to health care workers. Summing up all items leads to a total score of 4 to 20. The mean score above 10.89 was regarded as high patient anticipated stigma to health workers, while the score below 10.89 was regarded as low patient anticipated stigma to health workers [27].

### Data quality control

The English and Amharic versions of the dependent variable questionnaire were obtained from the validator of the tool; the validated tool was used. The semi-structured interviewer-administered questionnaire for independent variables was prepared in English first and then changed to the local language (Amharic) for the better understanding of the data collectors and the respondents, and then it was changed back to English again to check its consistency. Training was given for data collectors on the objective of the study, data collection tools and procedures, how to approach potential respondents, and how to maintain confidentiality. Pretesting on 5% of the sample size (21 participants) was done a week before the actual data collection period at Addis Alem General Hospital, located the other side of the city. The final data collection tool was refined based on the findings from the pretest. The collected data was carefully checked for completeness and consistency daily.

### Data processing and analysis

Epicollect5 was used to collect data, which was then directly exported to Statistical Product and Service Solution (SPSS) version 25 for further analysis. Descriptive statistics were used to describe the outcome and independent variables. Variables in the bivariate analyses at *P* < 0.25 were candidates for the multivariable logistic regression model. The adjusted odds ratio (AOR) with 95% CI and a *p*-value < 0.05 were used to announce the strengths and the factors significantly associated with the outcome variable respectively.

## Result

### Socio-demographic characteristics of participants

A total of 419 patients participated in this study, with a response rate of 99%. Nearly half of the participants were females (50.8%) and more than one-third (37.2%) of the participants were aged 26–35 years, with a mean age of 33.02 (± SD of 11.2). The majority of the participants (98.6%) were Amhara in ethnicity, nearly half (50.8%) of the participants lived in urban areas, and 26.0% were unable to read and write. 70.6% were Orthodox Christians, and one-third (30.1%) were farmers. Moreover, nearly half (47.5%) and more than one quarter (28.2%) of the participants were married and had a monthly income < 2000 birrs (with SD ± 3529 birr) respectively (Table 1).

**Table 1:** Socio-demographic characteristics of respondents’ care among patients with mental illness at TGSH and FHCSH, North West, Ethiopia, 2022 (*n* = 419)

Variables	Category	Frequency	Percent (%)
Sex	Male	206	49.2
	Female	213	50.8
Age	18–25	133	31.7
	26–35	156	37.3
	36–25	89	21.2
	≥ 46	41	9.8
Residence	Urban	213	50.8
	Rural	206	49.2
Ethnicity	Amhara	413	98.6
	Oromo	6	1.4
Religion	Orthodox	296	70.6
	Muslim	82	19.6
	Protestant	41	9.8
Marital status	Married	199	47.5
	Single	157	37.5
	Divorced	53	12.6
	Other^a^	10	2.4
Educational status	Cannot read and write	109	26
	Primary education	109	26
	Secondary education	109	26
	Diploma and above	92	22
Occupation	Farmer	130	31.1
	Private	128	30.5
	Gov’t employed	39	9.3
	Student	64	15.3
	Daily laborer	18	4.3
	Other^b^	40	9.5
Monthly income in the household(ETB)	< 2000	116	27.7
	2001–4000	118	28.1
	4001–6000	113	27.0
	> 6001	172	17.2

^a^separated, Widowed

^b^Housewife, Jobless, *ETB* Ethiopian Birr

### Clinical and psychosocial related characteristics

The majority of the participants (89.3%) were in follow-up and more than one-third (37.5%) of the participants attended their follow-up with a diagnosis of schizophrenia. Nearly 90.2% of the participants had 1–2 episodes and more than half (59.7%) had a duration of illness less than 24 months before the initiation of their treatment, with the median duration of illness of 24.00 months (Table 2).

**Table 2:** Clinical, social and service-related characteristics of respondents’ care among patients with mental illness at TGSH and FHCSH, North West, Ethiopia, 2022 (*n* = 419)

Variables	Category	Frequency	Percent (%)
Diagnosis	BPI disorder	73	17.4
	Schizophrenia	157	37.5
	MDD	124	29.6
	GAD	29	6.9
	Other^a^	36	8.6
Type of service	Follow up	374	89.3
	New	45	10.7
Duration of illness( Months)	< 24	250	59.7
	24–36	90	21.5
	≥ 36	79	18.9
Episode	1–2	378	90.2
	> 2	41	9.8
Family involvement	Yes	339	80.9
	No	80	19.1
CBHI	Yes	216	51.6
	No	203	48.4
Distance from the Hospital	< 10	102	24.3
	10–30	92	22.0
	> 30	225	53.7
Social support	Strong	192	45.8
	Moderate	127	30.3
	Poor	100	23.9
Perceived stigma	High	228	54.4
	Low	191	45.6
Shared decision making	Agree	213	50.8
	Disagree	206	49.2
Patients anticipated stigma toward health workers	High	228	54.4
	Low	191	45.6

(^a^Panic disorder, Schizophreniform, Dysthymia, Posttraumatic stress disorder) *BPI* Bipolar I disorder, *MDD* Major depressive disorder, *GAD* Generalized anxiety disorder, *CBHI* community-based health insurance

Nearly half of the participants had strong social support, and more than half of the participants had high perceived and anticipated stigma toward health workers (Table 2).

### Service-related characteristics

The majority of the participants (80.9%) had family involvement during their treatment. More than half of the participants came from a distance of more than 30 km and had community-based health insurance. Additionally, more than half the participants agreed with shared decision making during their treatment and nearly half of the participants had high medication adherence (Table 2).

### Level of perceived compassionate care

**Fig. 2:**
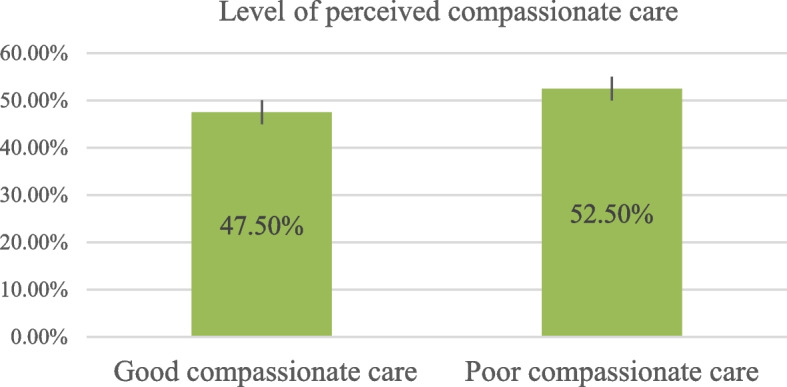
Level of perceived compassionate care among patients with mental illness at TGSH and FHCSH, North West, Ethiopia, 2022

The mean score of the participants was 76.74 (SD ± 21.74). Overall, 199 (47.5%) (95% CI = 42.6%-52.4%) of participants received good compassionate care at specialized TGSH and FHCSH (Fig. 2).

### Factors associated with perceived compassionate care

The results of this study revealed that residence, educational status, monthly income, duration of illness, family involvement in treatment, community-based health insurance, social support, shared decision-making, perceived stigma, and patient anticipated stigma towards health workers were significantly associated with compassionate health care at the bivariate level of analysis.

The multivariable analysis showed that residence, duration of illness, social support, shared decision-making, perceived stigma, and patient anticipated stigma towards health workers were significantly associated with perceived compassionate care. The odds of good compassionate care among patients with mental illness living in urban areas was 1.9 times (AOR = 1.90; 95%CI 1.08–3.36) more likely than among patients living in rural areas. The odds of good compassionate care among patients with a duration of illness less than 24 months was 2.68 times (AOR = 2.68; 95% CI 1.27–5.65) higher than patients with a duration of illness greater than 36 months. The odds of good compassionate care among patients with strong social support was 4.43 times (AOR = 4.43; 95% CI 2.16–9.10) higher than among patients with poor social support. The odds of good compassionate care among patients who agreed with shared decision-making was 3.93 times (AOR = 3.93; 95% CI 2.27–6.81) higher than those who disagreed with shared decision-making. The odds of good compassionate care among patients who had high perceived stigma from health workers was 2.97 times (AOR = 2.97; 95% CI 1.54–5.72) times higher than those who had low perceived stigma. The odds of good compassionate care among patients who had high anticipated stigma towards health workers was 2.92 (AOR = 2.92; 95% CI 1.56–5.48) times higher than among patients who had low anticipated stigma towards health workers (Table 3).

**Table 3:** Bi-variable and Multivariable binary logistic regression analysis showing the association between perceived compassionate care and associated factors among patients with mental illness at TGSH and FHCSH (*n* = 419)

Variables	Category	Perceived compassionate care		COR(95% CI)	AOR(95% CI)
		Good	Poor		
Residence	Urban	120	93	2.07(1.40,3.06)	1.90(1.08,3.36)*
	Rural	79	127	1.00	1.00
Education	Cannot read and write	43	66	1.00	1.00
	Primary education	52	57	1.40(0.82,2.40)	1.53(0.71,3.30)
	Secondary education	52	57	1.40(0.82,2.40)	1.09(0.49,2.39)
	Diploma and above	52	40	1.20(1.14,3.51)	1.63(0.67,3.95)
Monthly income(ETB)	< 2000	63	53	0.90(0.50,1.63)	0.96(0.39,2.35)
	2001–4000	49	69	0.54(0.30,0.97)	0.49(0.19,1.28)
	4001–6000	46	67	0.52(0.29,0.95)	0.66(0.28,1.58)
	> 6001	41	31	1.00	1.00
Duration of illness( months)	< 24	140	110	2.92(1.70,5.00)	2.68(1.27,5.65)*
	24–36	35	55	1.46(0.77,2.77)	2.28(0.96,5.45)
	> 36	24	55	1.00	1.00
Family involvement	Yes	169	170	1.66(1.01,2.73)	1.35(0.69,2.65)
	No	30	50	1.00	1.00
CBHI	Yes	112	104	1.44(0.98,2.11)	1.19(0.67,2.11)
	No	87	116	1.00	1.00
Social support	Strong	141	51	8.76(5.00,15.32)	4.43(2.16,9.10)**
	Moderate	34	93	1.16(0.63,2.12)	1.22(0.58,2.58)
	Poor	24	76	1.00	1.00
Shared decision-making	Agree	143	70	5.47(3.60,8.32)	3.93(2.27,6.81)**
	Disagree	56	150	1.00	1.00
Perceived stigma	Low	138	53	7.13(4.63,10.98)	2.97(1.54,5.72)*
	High	61	167	1.00	1.00
Patients anticipated stigma to health workers	Low	142	49	8.69(5.59,13.53)	2.92(1.56,5.48)*
	High	57	171	1.00	1.00

*CBHI* Community-based health insurance, *ETB* Ethiopian Birr

^*^p < 0.05 **p < 0.001

## Discussion

This study revealed that the level of good compassionate care at TGSH and FHCSH was 47.5% (95% CI = 42.6–52.4). This implies that compassionate care needs high attention as it is essential for quality health service [5] and 90% of patients switched to more compassionate health care professionals [24] that leads to good treatment outcomes [28].

This result was higher than a study finding in the North Showa, Ethiopia in outpatient service, where companionate care provision accounted for 38.8% [6]. The variation may be explained by the settings, the study population, and tool differences used in the studies. For instance, the study in the North Showa zone used 24 question items for non-psychiatric patients. Moreover, the study setting in this zone included both hospitals and health centers [18]. In contrast, only patients who visited for mental health services were included in this study, which used newly validated 12- item tools [19].

The result of this finding was in line with the result of the study done in Addis Ababa (48.0%) [18] on the health professionals' perspective at a health center and in North East Ethiopia (51.55%) on the patient perspective of compassionate care [7].

The level of good compassionate care in this study was lower than in America (53%) [8]. This higher proportion of companionate care provision in the USA might be due to differences in the healthcare development, study population and patient attitude towards mental health workers [18].

This finding was much lower than the study conducted in modern health facilities in Ethiopia (60.4%), the East Harerge zone, Ethiopia (71.7%), and North West Ethiopia (72.8%) The possibility of this difference could be the study setting, sample size, study population, instrument, socio-cultural differences, and analytical differences [10, 25, 26].

Patients living in urban areas were 1.9 times (AOR = 1.90; 95%CI 1.08–3.36) more likely to receive good compassionate care than those who lived in rural areas. This finding might be due to the availability of information about mental health service and different promotions on use of mental health services in urban, could help to increase patients' positive attitudes towards mental health workers. This finding was consistent with the study done in Addis Ababa [9].

This study indicates that patients with mental illness with a duration of illness of less than 24 months were 2.7 times (AOR = 2.68, 95% CI 1.27–5.65) more likely to get good compassionate care than those patients with a duration of illness greater than 36 months. This finding might be due to how the duration of illness affects the quality of care; if patients come early to clinic, mental health workers might be more satisfied and empathic to those patients than who came to clinic so late [27, 29].

This study showed that patients with strong social support were 4.43 times (AOR = 4.43, 95% CI 2.16–9.10) more likely to receive good compassionate care than those patients with poor social support. This finding supported by the study conducted in Northwest England and Ethiopia [6, 30]. This could be due to strong social support has being highly associated with good medication adherence, improved quality of life, and improved treatment outcomes therapeutic [31].

This study also indicates that patients who agreed with shared decision-making were 3.9 times (AOR = 3.93, 95% CI 2.27–6.81) more likely to receive good compassionate care than those who disagreed with shared decision-making. This may be because of shared decision-making between the patient and the supportive members including family and clinician is important to have good compassionate care that leads to a good treatment outcome [32, 33].

Patients who had low perceived stigma were 2.97 times (AOR = 2.97, 95% CI 1.54–5.72) more likely to receive good compassionate care than those who had a high perceived stigma. The possible justification might be perceived stigma could affect the level of therapeutic communication by inducing psychological distress among patients that leads them to receive low levels of compassionate care [22, 34].

Patients who had low anticipated stigma towards health workers were 2.9 times (AOR = 2.92, 95% CI 1.56–5.48) more likely to get good compassionate care than those who had high anticipated stigma towards health workers. This may be due to the fact that anticipated stigma hinders the level of good compassionate care and cultivating health-seeking behavior [10].

### Strengths and limitations of the study

To minimize bias, we used a standardized and pre-tested questionnaire. The study’s findings might be prone to a response bias due to the patients self-report, and they may not provide an objective measure of compassionate care. We included new patients who may not have history of contact with health workers before.

## Conclusion

Less than half of the patients with mental illness had received good compassionate health care in two hospitals, Northwest Ethiopia. Compassionate mental health care needs high attention as it is essential for quality health service. Factors including residency, duration of illness, social support, shared decision-making, perceived stigma, and patient anticipated stigma towards health workers were associated with level of compassionate care.

## Supplementary Information

**Additional file 1.** English version questionnaires.

## Data Availability

The datasets generated for this study are available on reasonable request to the corresponding author.
