# Supplementary material for: Perceived compassionate care and associated factors among patients with mental illness at Tibebe Ghion specialized and Felege Hiwot comprehensive specialized hospital, Northwest Ethiopia
Source: BMC Health Serv Res. 2023 Jun 17;23:650. doi: 10.1186/s12913-023-09665-4 (PMC10276910; doi:10.1186/s12913-023-09665-4)
Supplement: Supplementary file 1 — Additional file 1. English version questionnaires. [file 12913_2023_9665_MOESM1_ESM.docx]

English version questionnaires

Part I: Socio demographic Characteristics of patient respondents.

Instruction: For each of the following questions please circles the alternative that fit for respondent’s response.

| Code | Question | Answer category | Skip |
| --- | --- | --- | --- |
| 101 | Sex | 1. Male 2. Female |  |
| 102 | What is your age in completed years? | _____________________ |  |
| 103 | Residence | 1. Urban 2. Rural |  |
| 104 | Ethnicity | 1. 1. Amhara 2. Oromo 3. Other, specify ---- |  |
| 105 | What is your religion? | 1. Orthodox  2. Muslim 4. Other specify___  3.Protestant |  |
| 106 | What is your current marital status? | 1. Single 4. Separated  2. Married 5. Widowed  3. Divorced |  |
| 107 | What is your educational level? | 1. Cannot read and write  2. Can read and write  3. Grade 1-8 4. Grade 9-12  5.Diploma and above |  |
| 108 | \| Occupation \| \| --- \| | 1. Gov’t employed 4. Merchant  2. Private 5. Student  3. Farmer 6. Other/specify___ |  |
| 109 | Average Monthly income of the household in ETB |  |  |

Part II: Patient’s medical history

Instruction: This part of questionnaire deals with study participant’s information about their illness, hospitalization and frequency of visit.

| Clinical and service related factors | | | |
| --- | --- | --- | --- |
| Code | Question | Answer category | Skip |
| 201 | Full name of the diagnosis |  | To be filled from patient card |
| 202 | Duration of illness | _________ |  |
| 203 | Number of episode | ________ |  |
| 204 | Types of visit | 1. 1. New 2. 2. Follow up |  |
| 205 | Is your family involved during your treatment? | 1. 1. Yes 2.No |  |
| 206 | Have you community health insurance? | 1. 1. Yes 2.No |  |
| 207 | What is the distance from this hospital | _____________Km |  |

Part III: The Schwartz Center Compassionate Care Scale tool

Instructions: For the following questions please mark on 1 when the patient says the doctor (or other healthcare provider) not at all successful, mark on 10 when the patient says the doctor (or other healthcare provider is very successful and for neutral mark on 2,3,4,5,6,7,8 and 9.

| **Code** | | **Elements of compassionate care** | **1** | **2** | | **3** | | **4** | | **5** | | **6** | | **7** | | **8** | | **9** | **10** | | **Skip** | | | |
| --- | --- | --- | --- | --- | --- | --- | --- | --- | --- | --- | --- | --- | --- | --- | --- | --- | --- | --- | --- | --- | --- | --- | --- | --- |
| “Now, I would like to turn to an approach to treating patients known as compassionate health care that focuses on improving the relationships between doctors, nurses and other professional caregivers and patients and their families. Its particular focus is to improve the communication and emotional support that patients receive from their doctors, nurses and other professional caregivers.  On a scale of 1 to 10, where 1 is not at all successful and 10 is very successful, how successfully did your doctor (or other healthcare provider): | | | | | | | | | | | | | | | | | | | | | | | | |
| **301** | Show respect for you, your family, and those important to you | | | |  | |  | |  | |  | |  | |  | |  | | |  | |  |  |  |
| **302** | Convey information to you in a way that is understandable | | | |  | |  | |  | |  | |  | |  | |  | | |  | |  |  |  |
| **303** | Communicate test results in a timely and sensitive manner | | | |  | |  | |  | |  | |  | |  | |  | | |  | |  |  |  |
| **304** | Treat you as a person, not just a disease | | | |  | |  | |  | |  | |  | |  | |  | | |  | |  |  |  |
| **305** | Listen attentively to you | | | |  | |  | |  | |  | |  | |  | |  | | |  | |  |  |  |
| **306** | Always involve you in decisions about your treatment | | | |  | |  | |  | |  | |  | |  | |  | | |  | |  |  |  |
| **307** | Gain your trust | | | |  | |  | |  | |  | |  | |  | |  | | |  | |  |  |  |
| **308** | Consider the effect of your illness on you, your family, and the people most important to you | | | |  | |  | |  | |  | |  | |  | |  | | |  | |  |  |  |
| **309** | Comfortably discuss sensitive, emotional, or psychological issues | | | |  | |  | |  | |  | |  | |  | |  | | |  | |  |  |  |
| **310** | Express sensitivity, caring, and compassion for your situation | | | |  | |  | |  | |  | |  | |  | |  | | |  | |  |  |  |
| **311** | Spend enough time with you | | | |  | |  | |  | |  | |  | |  | |  | | |  | |  |  |  |
| **312** | Strive to understand your emotional needs | | | |  | |  | |  | |  | |  | |  | |  | | |  | |  |  |  |

**Part IV. The Oslo 3-items social support scale**

Circle or underline the correct answer that applies for you

| 401 | How easy can you get help from neighbors if you should need it? | Very easy(5) | Easy(4) | Possible(3) | Difficult(2) | Very Difficult(1) |
| --- | --- | --- | --- | --- | --- | --- |
| 402 | How many people are so close to you that you can count on them if you have serious problems? | None (1) | 1-2(2) | 3-5 (3) | 5+(4) |  |
| 403 | How much concern do people show in what you are doing? | A lot(5) | Some(4) | Uncertain(3) | Little(2) | No(1) |

Part V. The 9-item Shared Decision Making Questionnaire (SDM-Q-9)

| 601 | My doctor made clear that a decision needs to be made. | completely disagree(0) | strongly disagree(1) | somewhat disagree(2) | somewhat agree(3) | strongly agree(4) | completely agree(5) |
| --- | --- | --- | --- | --- | --- | --- | --- |
|  |  |  |  |  |  |  |  |
| 602 | My doctor wanted to know exactly how I want to be involved in making the decision |  |  |  |  |  |  |
| 603 | My doctor told me that there are different options for treating my medical condition. |  |  |  |  |  |  |
| 604 | My doctor precisely explained the advantages and disadvantages of the treatment options. |  |  |  |  |  |  |
| 605 | My doctor helped me understand all the information. |  |  |  |  |  |  |
| 606 | My doctor asked me which treatment option I prefer |  |  |  |  |  |  |
| 607 | My doctor and I thoroughly weighed the different treatment options. |  |  |  |  |  |  |
| 608 | My doctor and I selected a treatment option together |  |  |  |  |  |  |
| 609 | My doctor and I reached an agreement on how to proceed |  |  |  |  |  |  |

Part VI. Stigma Scale for Receiving Psychological Help

|  | Item | Strongly disagree (0) | Disagree (1) | Agree (2) | Strongly agree (3) |
| --- | --- | --- | --- | --- | --- |
| 701 | Receiving treatment for emotional or mental problems carries social stigma |  |  |  |  |
| 702 | It is a sign of personal weakness or inadequacy to receive treatment for emotional or mental problems |  |  |  |  |
| 703 | People will see a person in a less favorable way if they come to know that he or she has received treatment for emotional or mental problems |  |  |  |  |
| 704 | It is advisable for a person to hide  from people that he or she has been treated for emotional or mental problems |  |  |  |  |
| 705 | People tend to like less those who  are receiving professional help for  emotional or mental problems |  |  |  |  |

Part VII. Patients anticipated stigma to health workers

| S.no | Item | Very  Unlikely(1) | Unlikely(2) | Somewhat likely(3) | Likely | Very Likely |
| --- | --- | --- | --- | --- | --- | --- |
| 801 | Health care worker will be frustrated with you. |  |  |  |  |  |
| 802 | A health care worker will give you poor care. |  |  |  |  |  |
| 803 | A health care worker will blame you for not getting better. |  |  |  |  |  |
| 804 | A health care worker will think that you are a bad patient. |  |  |  |  |  |

Thank you!!!
